# Design and evaluation of a skin-on-a-chip pumpless microfluidic device

**DOI:** 10.1038/s41598-023-34796-3

**Published:** 2023-05-31

**Authors:** Marjan Mohamadali, Ali Ghiaseddin, Shiva Irani, Mohammad Amir Amirkhani, Mostafa Dahmardehei

**Affiliations:** 1 Department of Biology, Science and Research Branch, Islamic Azad University, Tehran, Iran; 2 Department of Chemistry, Michigan State University, East Lansing, MI USA; 3 Institute for Stem Cell Research and Regenerative Medicine, Tehran University of Medical Sciences, Tehran, Iran; 4 Department of Anatomical Sciences, Faculty of Medical Sciences, Tarbiat Modares University, Tehran, Iran; 5 Skin and Stem Cell Research Center, Tehran University of Medical Sciences, Tehran, Iran; 6 Department of Plastic and Reconstructive Surgery, Burn Research Center, Iran University of Medical Sciences, Tehran, Iran

**Keywords:** Biotechnology, Health care, Medical research

## Abstract

The development of microfluidic culture technology facilitates the progress of study of cell and tissue biology. This technology expands the understanding of pathological and physiological changes. A skin chip, as in vitro model, consisting of normal skin tissue with epidermis and dermis layer (full thickness) was developed. Polydimethylsiloxane microchannels with a fed-batched controlled perfusion feeding system were used to create a full-thick ex-vivo human skin on-chip model. The design of a novel skin-on-a-chip model was reported, in which the microchannel structures mimic the architecture of the realistic vascular network as nutrients transporter to the skin layers. Viabilities of full-thick skin samples cultured on the microbioreactor and traditional tissue culture plate revealed that a precise controlled condition provided by the microfluidic enhanced tissue viability at least for seven days. Several advantages in skin sample features under micro-scale-controlled conditions were found such as skin mechanical strength, water adsorption, skin morphology, gene expression, and biopsy longevity. This model can provide an in vitro environment for localizing drug delivery and transdermal drug diffusion studies. The skin on the chip can be a valuable in vitro model for representing the interaction between drugs and skin tissue and a realistic platform for evaluating skin reaction to pharmaceutical materials and cosmetic products.

## Introduction

Due to several benefits, human skin models draw more and more attention in research works. The skin is the largest human organ which is not only the first defense line of the body, but it also has the duty of featuring human appearance beauties. Being the outermost layer of the body, the skin is exposed to mechanical, (bio) chemical, or biological environmental effects which cause dermal disease or damage^1,2^. On the other hand, several internal diseases change the skin appearance. In recent decades, transdermal drug delivery has added to the duties of the skin^1^.

Depending on the research work, the approach to preparing human skin equivalents varies. Skin is the largest organ that coats the surface of the body. It has many functions, including regulating heat, conducting physical sensations, and acting as a mechanical barrier (to protect the body against the invasion of microorganisms and harmful environmental factors such as radiation, mechanical damage, and heat and chemical burns). Skin tissue is made of three major layers, namely the epidermis, dermis, and subcutaneous layer^3^.

With the approval of the Animal Prohibition legislation for cosmetic and chemical ingredient testing, there is a need for alternative 3D models to replace animal testing^4,5^.

The lack of similarity between human and animal skin and the ethical reasons for using animal models have fueled studies on designing new skin models that are more similar to real skin^6^.

The development and evolution of skin tissue in vitro have led to the development of 3D skin models. The physiological response of cells in these models in terms of biochemical signals and structural and mechanical properties should be like actual skins^7^. Advanced models are also needed to precisely demonstrate drug-cell interactions.

Scientists use standard cellular tissue technologies and animal models in the preclinical stages^8,9^. Clinical trials are one of the last stages of drug production and the high failure rate at this stage is because the results obtained in cell-based experiments on animal models and in-vitro, which fail to represent the cell's response to the drug in vivo^10–13^.

However, these models are not yet effective in mimicking all aspects of human skin function and complexity, mainly due to a lack of cell variety, innervation, and vascularization^14^.

Microfluidics can create better platforms for examining skin equivalents under a controllable environment^15^.

Given that the combination of different cell types with a wide variety of structural proteins is needed to build skin, using Ex-vivo human skin biopsies looks very appealing and is expected to return the closest model to the real skin. In this regard, for several decades, the Franz apparatus was the most prevalent experimental setup using human living skin biopsies^16^.

There are two major obstacles to designing experiments using the Franz apparatus. The first is finding enough skin, and the second is using a significant amount of medium in the apparatus dilutes all the secreted metabolites from the skin or transferred material in transdermal drug delivery studies^1,2^.

Up until now, different microfluidic systems have been designed and performed. Many of these models have enhanced our understanding of sciences such as physiology, pharmacology, and toxicology. The organ-on-a-chip concept is a combination of cell culture techniques and protocols and fluid systems in microliter volumes to analyze the normal functions of human organs^17^.

Through miniaturizing the experimental setup, the needed skin surface is reduced by several folds. In addition, through the micro channels and micro fabrication, nutrients can be transferred to the skin cells more efficiently which decreases the amount of medium. In case of having a high volume of skin and medium, applying a microfluidic device seems optional but limited resource of living human skin, in one hand, and needs to trace of very little number of active molecules in the medium on the other hand elaborate skin-on-a-chip as a vital choice in compare with traditional methods. A microfluidic device is the main solution to overcome the obstacles^18–23^.

In the following study, the physicochemical and biological differences were compared between a human skin biopsy in macrosystems (traditional culture method) and when the skin is mounted onto a micro vascularized microbioreactor (µBR). In the end, an Ex-vivo human skin model on a chip was introduced that can be highly recommended for drug screening tests and transdermal drug delivery studies.

## Results and discussion

There are several commercial reconstructed skin models with limitations. For instance, they cannot mimic the nature of skin^24^.

Scaffolds, in bioartificial 3D constructs aimed to resemble skin, function as ECM. They degrade rapidly and have a shortened lifetime, which notably decreases their applicability over time^2^.

Most Human Skin Equivalents models have a simplified bi-layer-setup, including epidermal and dermal layers with simplified cellular such as keratinocytes and fibroblasts respectively. These setups cannot mimic micro architecture that features cell–cell and cell–matrix interaction, diverse cellular composition, and dermal-epidermal signaling events^25^.

In addition, due to the natural differences between animal and human skin physiology ^26^, the results from animal model experiments, especially in the field of transdermal drug delivery, could not predict human skin responses^27^. Thus, many of the potential drugs, which show promising results in animal model studies, fail in clinical trial steps^28^.

One drawback is the lack of blood vessels in these models that results in limitations in dynamic transport of nutrients and real-time measurement^29^.

Living human skin on a chip model represented here is a reliable candidate to overcome the limitations because it creates a skin microenvironment as close as possible to human in vivo conditions. These models provide a reaction between skin and ECM in microfluidic platforms. Due to the dynamic flow rate in microfluidic platforms, it is capable of mimicking microvasculature and therefore it is capable of analysis of dynamic transdermal drug delivery^29^.

A novel skin-on-chip device was introduced with an innovative microfluidic design that enabled the maintenance of normal skin tissue for a long time without using any support materials. Nutrient mediums pass through the microfluidic channels. Two plexiglasses seal PDMS layers prevent any media leakage. The flow of media via microchannels provides a continuous supply of nutrients and removal of waste products through outlets resembling the role of blood vessels in actual human skin tissue. The structural integrity of skin samples cultured in the microsystem was tested in comparison with a macro condition and permeability of skin samples.

### Design and operating conditions of skin chips

**Figure 1:**
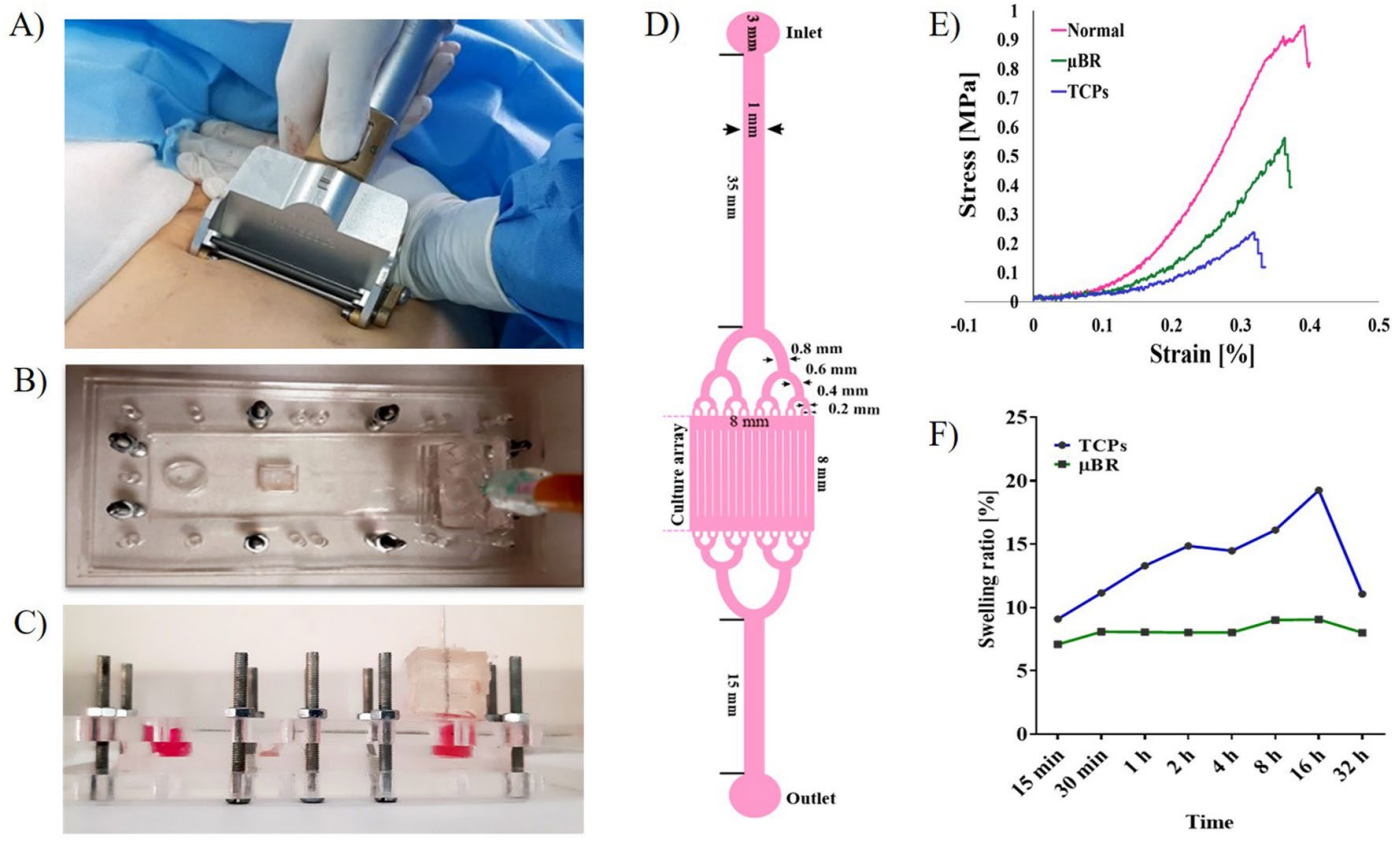
Skin graft images (**A**) Full-thickness skin graft harvested by microtome from a donor. The microfluidic device. (**B**) Image of a top view of a skin-on-a-chip device (For more details see supplementary 1). (**C**) Picture of the assembled skin-on-a-chip device in vertical display. The two-layer PDMS between two-layer polystyrene sheets tightened with screws. (**D**) Top schematic of the skin-on-a-chip pattern. (**E**) Stress–Strain curve of the normal skin cultured in TCPs and µBR. (**F**) The graph of swelling skin behavior ratio of the normal skin cultured in TCPs and µBR during 32 h.

The main frame of the skin chip model was prepared using PDMS. The chip consists of two layers that are covered by two polystyrene sheet layers. The bottom PDMS layer consists of microfluidic channels, a two-reservoir chamber, and a chamber for cultured skin tissue. The medium was supplied via a serum tube, connected to the bottom channels and waste removal was performed through an outlet. Figure 1C shows the image of the actual chip in vertical display and Fig. 1D shows the schematics of the skin on chip. The existence of the parallel micro channels in the skin chamber prevents any occlusion that may occur; moreover, it helps with an equal distribution of the media. The skin chamber is an 8 mm square which will be implanted with 1 cm^2^ of full thickness human skin biopsy^21,30,31^.

Current skin equivalent (SEs) models can mimic a very specific part of skin tissue and they are used for toxicology studies of drugs or cosmetic products' effect on a very specific layer of skin^32^. However, these models cannot mimic the dynamic of the full thickness of human skin conditions. Therefore, it is necessary to mimic native human skin tissue structure and functions to create the skin on chip model resembling normal-like-skin physiological conditions. To study cell–cell interactions, their responses to mechanical force, and various physical and chemical factors, the skin on a chip and the same skin samples in a traditional culture plate were studied. The results, in almost all the experiments, showed a significant decay in the tissue cultivated in the macro system in comparison with the semi-batch perfusion microfluidic system. Hence, the development of skin-on-chip models has prominent advantages compared to available options, such as portability, faster preparation, cost-effectiveness, and enhancing the efficiency of drug research^2^.

Various studies have demonstrated that SEs maintenance needs supplementary materials; however, we showed skin tissue samples can be kept alive without any expensive growth factors, in presence of a perfusion flow regimen.

The perfusion flow regimen was a renewing fed-batch that injected 500 µL of the medium every 8 h. The discharged materials were collected for further studies such as mass transfer measurements or glucose consumption which is not included in this article^31^.

The focus of the study was on designing a skin model with microvascular channels by exchanging sufficient nutrients and oxygen that leads to long-term physiologically conserved tissue survival which is detailed in cell viability section of the results and discussion.

### Physical and mechanical properties of the skin model

The skin sample was cultured with a specific size (0.8 cm^2^) on µBR and the device was designed in such a way that the rate of stretching and shrinkage was kept constant during the test. This was not possible in TCPs condition, so the skin samples were floated on the medium.

As shown in Fig. 1E, the tensile result for the skin sample in the µBR decreases by about 30% after one week while, the mechanical strength for TCPs is down to 70% after 1 week^33^. The mean of three repeats of identical donor samples is reported in the graph, it is noteworthy that the standard deviation was less than 6% between the three runs.

The swelling ratio diagram showed that the skin samples in the TCPs group had a higher adsorption rate due to the high amount of medium than the µBR sample group (Fig. 1F). The results are consistent with the read-out from the tensile strength test. In all traditional setups, such as the Franz apparatus, included skin samples are in contact with or submerged in a culture medium. As is shown in Fig. 1F, when a normal skin sample comes in contact with several times larger volume of an aqueous solution, the skin absorbs water even if the medium is an isotonic solution. ECM has great potential to retain liquids. Based on these results, water retention is highly proportional to the amount of available liquid medium^33^. The swelling graph shows 7% water adsorption in µBR group after 16 h, whereas TCPs samples adsorb about 20% at the same time which is about threefold higher which can lead to dramatic changes in all aspects of the skin properties. The swelling graph shows 7% water adsorption in µBR group after 16 h, whereas TCPs samples adsorb about 20% at the same time which is about threefold higher which can lead to dramatic changes in all aspects of the skin properties. The mean of three repeats from identical donor sample is reported in the graph, it is noteworthy that the standard deviation was less than 8% between the three runs.

One of the main characteristics of the skin is moisture level control. Moisture in the skin plays an important role in maintenance, metabolism, enzymatic activity, mechanical properties, and appearance and eventually plays a functional and protective barrier. Air humidity and underlying skin moisture are the only sources of skin moisture^34^. The corneum layer protects other layers of the epidermis, in addition preventing water loss and entry of external factors. Thus, this layer shows the greatest resistance in mass transfer. Given that in macro setups the skin samples are drenched with an aqueous solution, and the interstitial space of the cells are filled with hydrophilic medium, the setups facilitate mass transfer which is far from the real characteristics of the skin.

An interesting observation was a significant drop after 32 h in the capability of water retention which can be related to the steep slope of degradation of skin matrix, which needs further studies. No matter what the reason is, applying the skin biopsy in a system with plenty of available liquid medium leads to adsorbing up to 20% liquid which causes swelling. Swollen skin mass transfer coefficient is definitely not the same as normal skin.

Based on this observation it seems that using microfluidic systems is not just an option to reduce costs. In other words, being drenched in liquid medium in TCPs facilitates swelling which deforms and even deteriorates the skin structure which is in consistent with histological results and electron micrographs of the sample (the results will be represented later in this article). Several histological studies have been carried out^35–38^ in-vitro skin model samples which could be misleading due the sample deformation because of swelling.

### Cell viability within the skin chip

One of the key points for the successful operation of the skin chip model is the efficient transport of nutrients from microchannels to the dermis and epidermis layers of the skin. Skin tissue samples' behavior such as proliferation and viability were analyzed by MTT assay and Acridine-Orange (AO) staining in µBR and TCPs conditions and were compared between two groups for 7 days.

**Figure 2:**
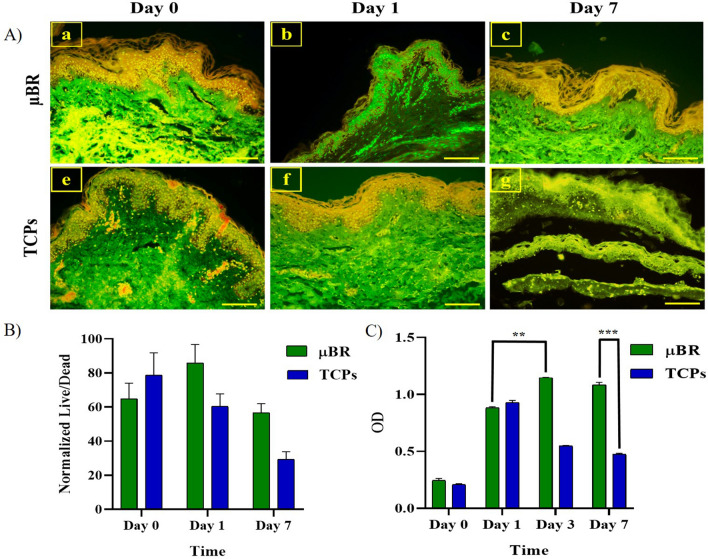
(**A**) A fluorescent microscopic image of live full thickness skin tissue (green fluorescence) in µBR (a, b, c) and TCPs (e, f, g) on days 0, 1, and 7. (Scale bar 300 μm). (**B**) Quantified graph of viable full thickness skin cells by AO staining during 7 days. (**C**) Viability results of full-thickness skin tissue samples cultured in two groups (µBR and TCPs) for 7 days using MTT assay, (*P*-values < 0.01 (**) and *P*-values < 0.001 (***)).

To confirm the viability of skin samples in two groups, an MTT assay was performed. According to the chart (Fig. 2C), the viability of TCP samples was reduced in comparison with µBR samples after seven days.

As shown in the MTT chart (Fig. 2C), the µBR can recover the biopsies as well as incubate the tissue in a conventional condition (i.e. submerging tissue in the supplemented nutritional medium). After 24 h both groups showed significant changes in their living signals. The living optical density signal in the MTT assay on day 0 is very low because the tissue is kept in cold PBS to reduce the metabolic activity to a minimum possible and increase the viability until transferring the tissue into µBR. Therefore, after one day of cultivation, the viability signals recovered to more than 0.9 in both groups. However, after the third day of culture, a steep drop in viability appeared which can be addressed through the lack of perfusion in this group.

These results are consistent with the data obtained from AO staining. AO dye easily penetrates living cells and intercalates into DNA. If the AO dye binds to dsDNA, it produces a green color in fluorescent microscopy, while it produces red–orange color if it binds to ssDNA or RNA. The green color confirmed viable cells^31,33^. As pictured in Fig. 2A, the portion of fluorescent green surfaces in µBR samples (day seven) was higher compared to TCP samples (day seven), which confirmed the survival of the samples cultured in this condition. Samples' viability decreased on day seven compared to day zero in both groups (Fig. 2B).

Figure 2Ag shows that disintegrated tissue remained after seven days of culture in TCPs, which is consistent with the results of mechanical properties and swelling ratio tests. Hydrolysis of ECM and loss of dead cells from the horny layer through digestion can explain the phenomenon.

### Histological analysis

**Figure 3:**
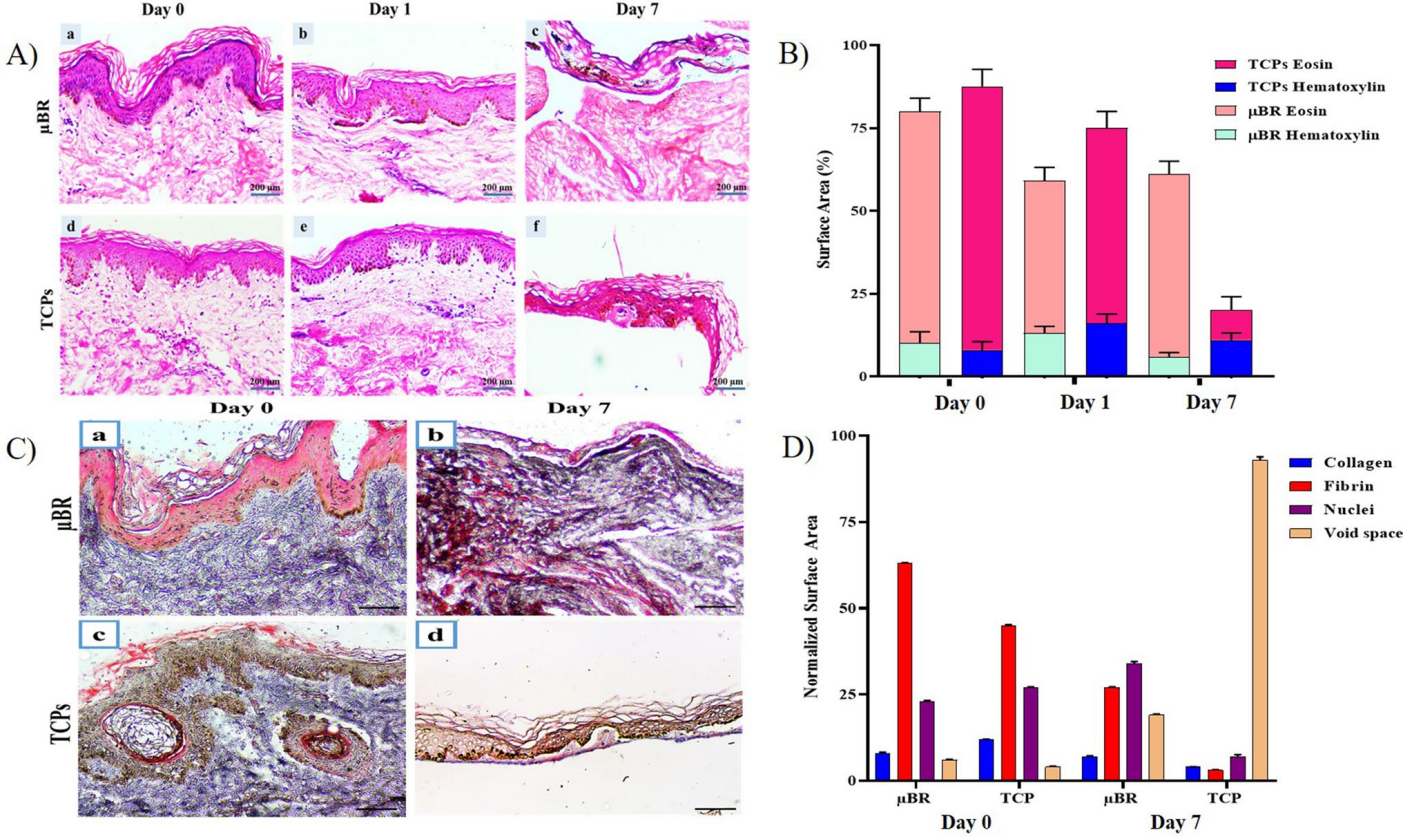
Skin histology. (**A**) H&E staining microscopic images of the skin tissue samples cultured in µBR (a, b, c) and TCPs (d, e, f) on days 0, 1, and 7. The nuclei of cells stained purplish blue, and the cytoplasmic components stained pink. (**B**) Statistical analysis of the H&E staining results during 7 days. (**C**) Microscopic image of the skin tissue samples stained with Masson’s Trichrome staining in a, b) µBR and c, d) TCPs on days 0 and 7. The collagen matrix protein in dermis layer stained blue, fibrin in pink, and nuclei in black (**D**) Quantified graph of dermis component stained by Masson's Trichrome.

To confirm the observation of fluorescent microscopy, the structural integrity of the skin tissue samples cultured in the µBR, and TCP was examined through H&E staining. H&E staining was used to identify the different skin layers^39,40^. According to Fig. 3A, the structure of skin tissue in µBR, unlike the sample cultured in macro conditions (TCP), was not changed after seven days. The TCP samples showed that the stratum corneum was still attached to the viable epidermis and the dermis layer was removed after seven days. The thickness of the skin layer samples was measured using Image J software (Fig. 3B). The obvious results from the normalized numerical analysis showed tissue loss in TCPs after seven days of culture. According to Fig. 3B, the ratio of nuclei to the rest of the tissue is about 15% except in the TCPs group on day seven where the nuclei proportion jumped to about 50%. This shows that only live cells can stay in place and dead cells or other connective cells or proteins decay through hydrolysis or digestion.

After seven days in µBR, there was about 20% tissue loss, while all three layers of the skin were distinguishable and morphologically looked like normal skin.

### Histological analysis using Masson’s Trichrome staining

The skin is a complex structure made of a combination of different cells in a pool of a well-organized extracellular matrix (ECM). Masson’s Trichrome staining was developed to study connective tissue and ECM. The stain is capable of pronouncing the difference between collagen matrix protein in blue, fibrin in pink, and nuclei in black^39–41^. The amount of collagen/fibrin in skin samples in two groups was measured using Image J software (Fig. 3D).

Epidermal and dermal layers were clearly visible in the skin samples cultured in µBR on day 0. The thickness of the epidermis layer and the pigments were in normal condition. Inflammation was low and collagen fibers could be seen in all parts of the dermis layer (Fig. 3Ca). In µBR, the texture of tissue and the conformational arrangement of the dermal tissue was mostly the same, comparing the tissue at 0 and 7 days. After seven days, the cells were alive on most of the surface of the skin (Fig. 3Cb). It should be noted that it does not mean there is no cell death; the dead cells were digested during perfusion and removed as cell debris.

The epidermis and papillary layers were visible in the TCPs samples on day zero. The epidermal layer had a good thickness. The granulosum portion contained dark pigments, and melanin was visible in the brown basal layer (Fig. 3Cc). On the contrary, Masson trichrome staining showed that the texture of tissue changed during the seven days of culture, which can indicate that only fibroblast cells could remain alive and proliferate in this condition (Fig. 3Cd).

The cells were alive in the TCP sample for seven days; however, the tissue structure deteriorated. The easiest noticeable observation was a massive loss in soft tissue parts like fat granules or hypodermis layer which remains a void space in the tissue structures. Figure 3D shows the jump in void spaces on day seven of TCPs. Although in µBR a depreciation and color change in ECM protein was visible on day seven and the skin morphology and appendages were all in place.

The properties of both stratum corneum and viable epidermis were influenced by environmental conditions, such as relative humidity and temperature. The cellular membranes in the stratum corneum were thickened so a larger amount of keratin was present, and the water content decreased. Thus, its mechanical stiffness and strength are suggested to be even higher.

### SEM imaging

**Figure 4:**
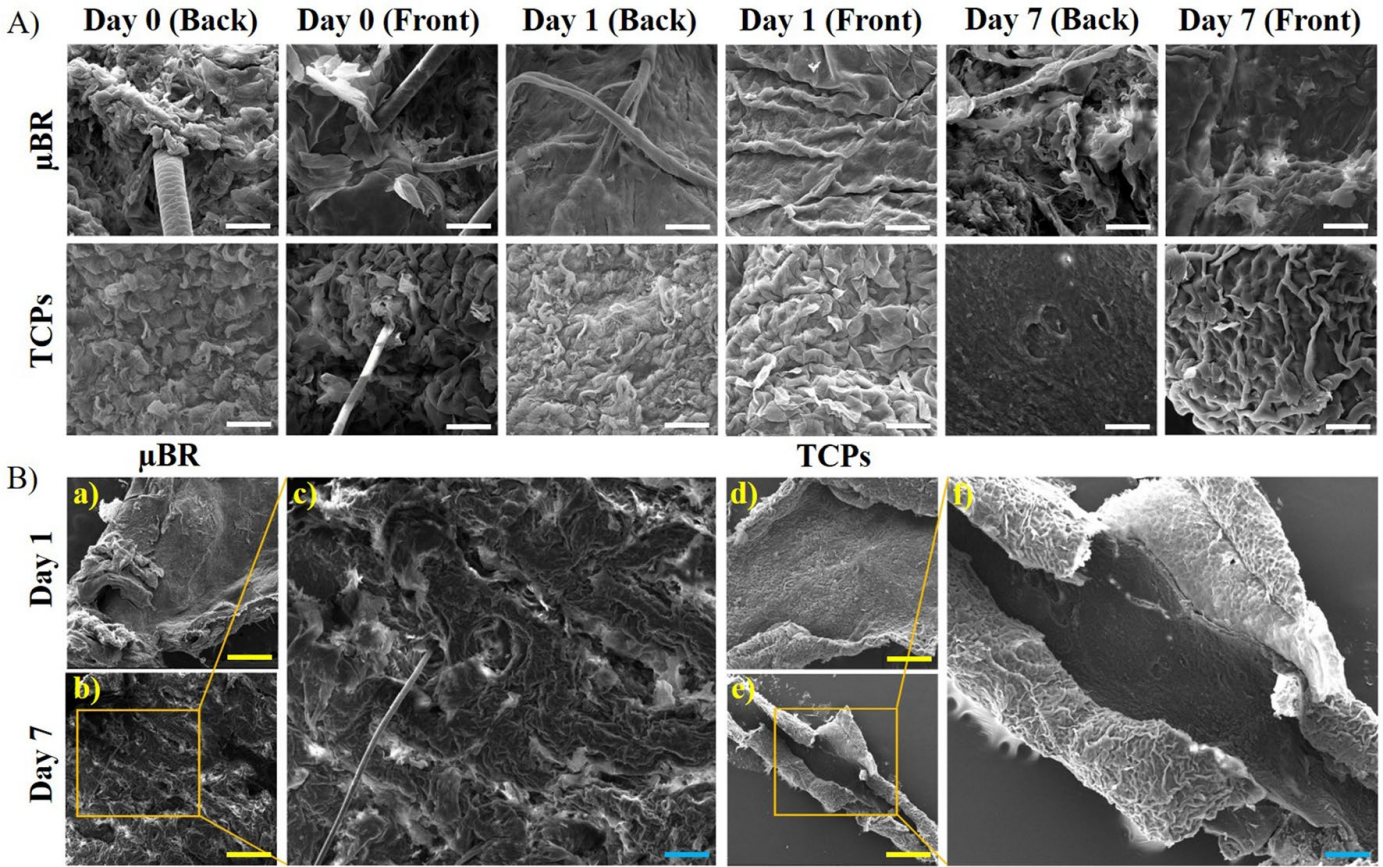
(**A**) Scanning electron Micrographs of full-thickness skin tissue samples cultured in µBR and TCPS on days 0, 1, and 7. (**B**) Skin tissue samples cultured in a, b) µBR and d, c) TCPS on days 1 and 7. c) The arrow points to an enlarged part of the skin samples in µBR situation after 7 days. f) The arrow points to an enlarged part of the skin samples in TCPs that shows the dermis layer lost. (The white, yellow, and blue scale colors refer to 100 μm and 1.0 mm, and 500 μm respectively).

Morphology of the basal and outermost surface of skin samples after being cultured in two groups (µBR and TCPs) were assessed by SEM, in 0, 1, and 7 days (Fig. 4A).

Because of swelling and protein hydrolysis^42^, the epidermis was detached from the stratum corneum layer in the TCPs sample images on day seven, which indicated that the skin was in slippage status and deterioration (Fig. 4Bf). However, in µBR samples, even the structure of hair follicles was normally preserved after seven days (Fig. 4A,B). The dermis is located under the epidermis layer and with a membrane between them. The strength and flexibility of this area are due to the presence of collagen and elastin fibers. Type I and type III Collagen are abundant in this layer^43^. As shown in Fig. 4C, the skin lost its connective interface membrane, probably due to protein hydrolysis or digestion, which caused dermis-epidermis detachment. Moreover, the individual ECM fibers became visible in TCPs samples after seven days, due to the washing of the digested materials (Fig. 4Bd).

In comparison with TCPs, the integrity between the layers and the overall surface of the tissue in µBR looked almost intact.

### RT-qPCR

**Figure 5:**
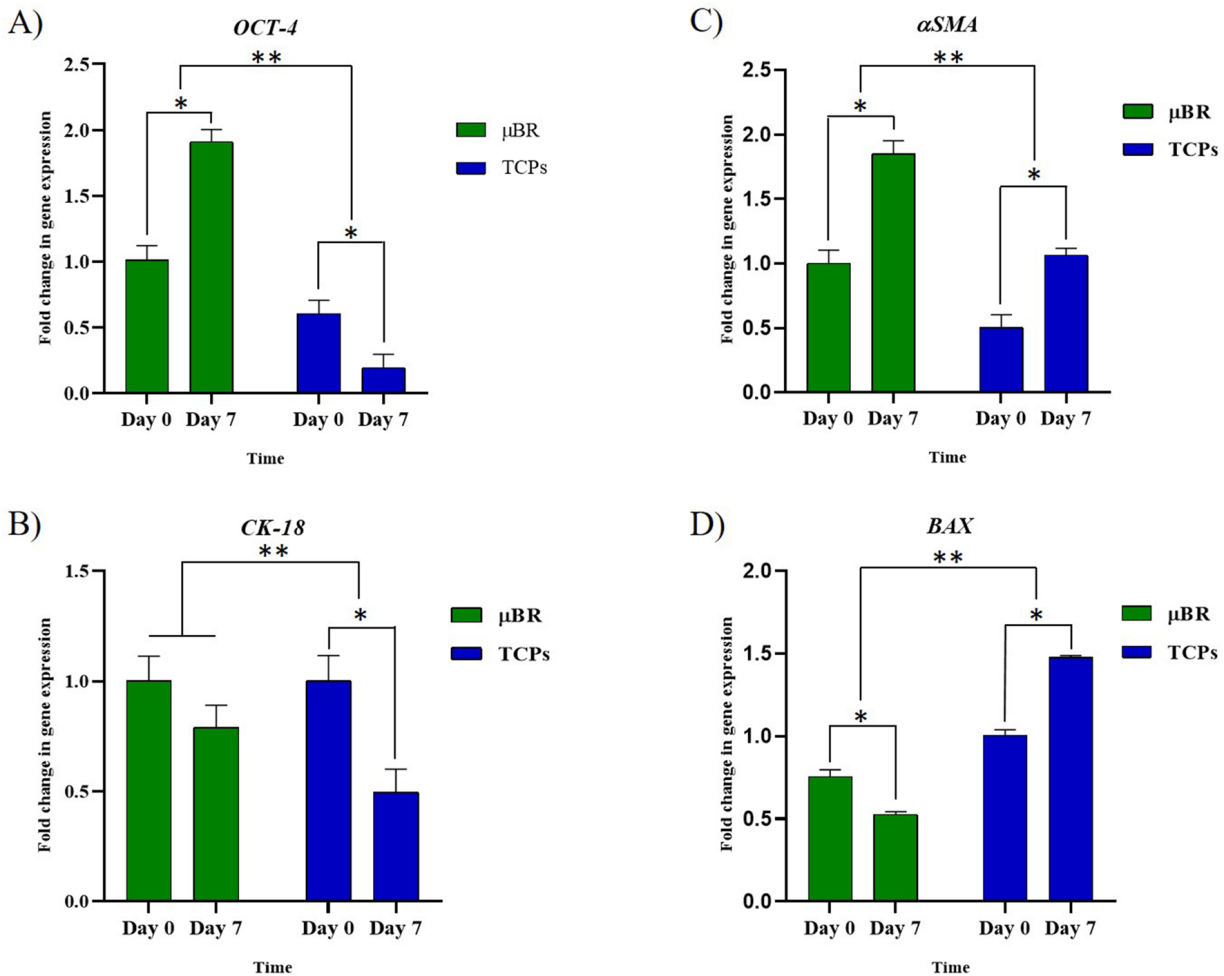
The RT-qPCR analysis. The expression levels of the *β-actin* (as an internal gene), *OCT-4* as a Proliferation gene, *CK18* as keratinocyte markers, *α-SMA* as a fibroblastic marker*,* and *BAX* genes as an apoptotic marker were detected by Real-time PCR in TCPs and µBR skin samples on days 0 and 7. The fold changes of the analyzed RT-PCR by REST software, (*P-values < 0.05 and **P-values < 0.01).

RT-qPCR was performed to evaluate the expression changes in the genes of interest in the skin samples for seven days (Fig. 5).

There was a significant change in *OCT-4* expression in µBR compared to TCPs from day zero to day seven. It can be explained that the remaining cells on skin tissue were more proliferative after seven days and there were fewer deaths due to a better physiological condition. Therefore, the stem cells proliferate faster in µBR compared to TCPs. Up- regulation of the expression level of *OCT-4* gene was observed in µBR samples compared to TCP samples after seven days (*P-values < 0.05).

As for *α-SMA* as the fibroblasts marker, the results proved there was no complete physiologic condition to differentiate stem cells due to low access to nutrition in both µBR and TCPs conditions. Up-regulation of *α -SMA* gene in the µBR samples after seven days was confirmed (*P-values < 0.05). Increased expression of fibroblastic marker (*α-SMA*) is the native skin response to maintain its integrity which can confirm increase in *OCT-4* gene expression. Downregulation in the *CK-18* gene expression, the keratinocyte marker, in the µBR samples revealed the proliferative marker in favor of fibroblastic cell proliferation. On the other hand, the overall transcriptomics level in the µBR group was remodeled from dermal to fibrotic.

There were, in specific keratinocyte markers (*CK18*) in the µBR, which was a sign of maintenance of the mimic of physiological condition compared to TCPs that have a significantly lower expression of *CK 18* from day zero to day seven. This result can confirm the *OCT-4* low expression in TCPs. (*P-values < 0.05).

The significant increase of *BAX* expression in TCPS indicates the activation of the programmed cell death. Due to the excision of the skin tissue, the lack of nutrition, and temperature shock that triggered the apoptosis pathway, there was a significant decrease in apoptotic marker (BAX) expression in µBR after seven days.

Altogether, we can suggest there are two levels in ex-vivo tissue treatment, at the first level the goal is to keep the tissue alive. At the second level, the aim is to keep the tissue in an enhanced condition in which tissue behavior represents its desirable physiological manner. The results of RT-qPCR showed that the conditions of TCP samples could not even provide the first level of the culture medium. On the other hand, the tissue in μBR is kept alive and there is a reasonable survival condition; however, the fibrogenesis marker of rapid growth showed that the μBR condition was not suitable enough to respond to the second level of treatment.

### Immunohistochemistry analysis

The expression of skin protein markers like CYTOKERATIN-18, VIMENTIN, ECADHERIN, and PAN-CYTOKERATIN were examined by immunohistochemistry (IHC). PAN-CYTOKERATIN is a marker for differentiating epithelial and mesothelial cells from mesenchymal cells in skin tissue^44^. One of the cytoskeletal proteins in keratinocytes is CK18 which is the main intermediate filament family member^45^.

E-CADHERIN has an important role in the morphogenesis of skin tissue. E- CADHERIN, which is a membrane protein, plays an important role in the organization and maintenance of skin epithelial cells. Epithelial tissue acts as a defense barrier against the external environment, and it is therefore subjected to damage and physical forces^46^.

VIMENTIN was found in the dermal layer of the skin samples. The biological impact of VIMENTN is attributed to the loss of fibroblast contractile capacity and expression of mesenchymal stem cell^47^.

**Figure 6:**
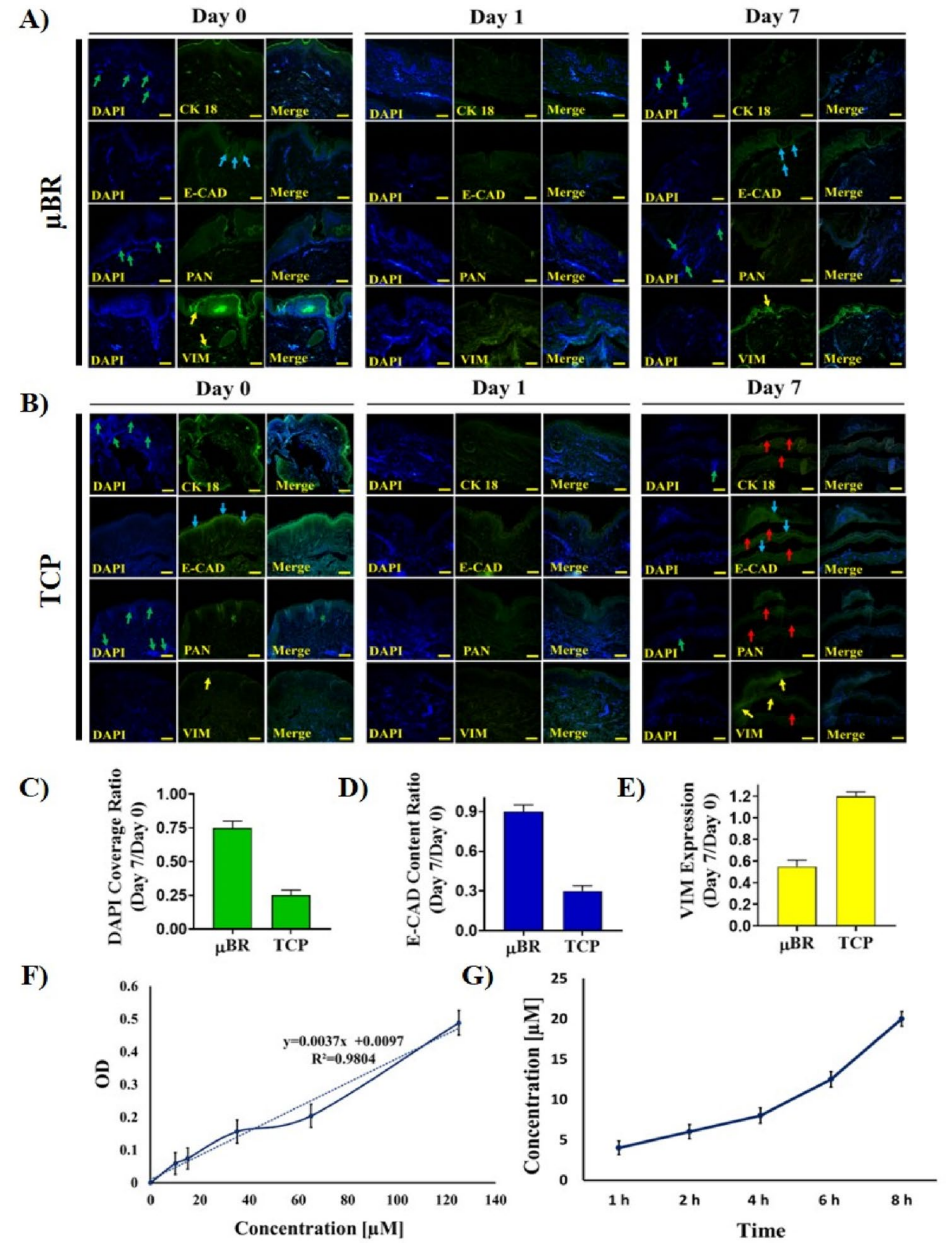
Immunohistochemically analysis. (**A**, **B**) Sections of 5 μm were prepared and stained for CYTOKERATIN-18, E-CADHERIN, PAN-CYTOKERATIN, and VIMENTIN proteins in two groups (µBR and TCPs) on days 0, 1, and 7. Cell nuclei stained with DAPI (blue) and shown by green arrows. Expression of E-CAD and VIM proteins stained by conjugated FITC antibodies, shown by blue and yellow arrows respectively. The red arrows point to regions showing loss of dermis layer in TCP groups (Scale bar 400X). (**C**–**E**) Quantified graph of DAPI coverage and E-CAD and VIM protein expression ratio during 7 days respectively. (**F**) Calibration curve for the estimation RA release at 340 nm by using UV–VIS spectrophotometer. (**G**) Amount of RA transferred through the skin sample cultured in µBR at different time points after administration. Line indicates real-time transferred drug concentration in µM during 8 h.

As illustrated in Fig. 6A,B, the green arrows show that the intensity of DAPI staining has declined to ~ 25% in TCPs groups in seven days in comparison to 75% of μBR. DAPI represents the number of nuclei in tissue. Hence, the cell density on cultured tissue in TCPs decreases to about one-third in comparison to the μBR group in seven days. This result is consistent with H&E study and also with the fluorescent image of the tissue in TCPs group on day seven, which is shown in Fig. 6B (Day 7) by red arrows. The green light intensity of E-CADHERIN in the μBR group remained at 90% of the level of day 0 which shows the ability of the device to keep the tissue in physiological condition (blue arrows in Fig. 6A). On the contrary and in TCPs group, not only the surface covered with cells is reduced but also the green light intensity of the E-CADHERIN marker shows a deep fall which results in a one-third decline in E- CADHERIN marker which is the main marker of skin epithelial in this study (blue arrows in Fig. 6B, day 0, 7). Finally, the VIMENTIN marker in TCPs shows a jump in TCPs which can be interpreted as the remaining of just mesenchymal stem cells in the TCPs culture. In fact, through elimination and washing of the other cells in TCPs during seven days of culture the mesenchymal stem cells which are capable of proliferation in this condition are pronounced. The changes in the expression of essential markers of the images of µBR samples in the seventh day were at least 33% less than that of the other groups (Fig. 6C–E). However, due to the loss of the major part of the dermis layer in TCPs samples, the expression of E-CADHERIN, CK18, PAN CYTOKERATIN, and VIMENTIN proteins decreased (Fig. 6C–E).

### Transport inside the skin on the chip

Skin-on-a-chip platforms are used for testing cosmetics materials as an alternative for replacing animal models. Furthermore, these platforms have also been utilized for drug screening not only for skin related pharmaceuticals but also for side-effect study of other systemic drugs reactions on skin^9^. However, transdermal drug delivery is a major advancement in the pharmaceutical industry which is not well developed yet. Every day a new drug carrier platform is introduced by research teams, which needs to be characterized in terms of active materials mass-transfer through the skin. Even one carrier platform for checking different active materials in a wide range of concentrations requires numerous skin models, which is very expensive. With an animal model, on the other hand, the results are not accurate enough for human skin applications.

Using this novel skin-on-a-chip device, it is possible to perform 400 runs using just 20 cm by 20 cm of human skin donated from a brain-dead patient or deceased person. The µBR is easy to run and real-time sampling is close to in-vivo concentration. With a proper experimental design methodology, 400 available skin devices should be more than enough for mass-transfer characterization, cytotoxicity, side-effect study and drug effectiveness evaluation for one drug/platform.

One of the key criteria for a successful design of skin-on-a-chip model is the efficient transport of nutrients from the bottom channels to the epidermis and dermis layer. There are several advantages in using this setup along with the very close imitation of real human skin conditions. One of the advantages is the microliter amount of the medium in-residence volume of the µBR. Using this advantage, even a very small amount of components which pass through the skin will be diluted in a microliter scale of medium which returns a detectable concentration. In other words, by using the microfluidic system, the limit of detection in the transdermal diffusion measurements enhances^4,16,18,34^.

To show the advantages of the device in real-time mass-transfer study, we tried to show that the fabricated µBR is well suitable for real-time monitoring of chemical materials (retinoic acid (RA)) diffusion measurement through the human skin. The results of RA delivery as mass transfer molecule through the cultured human skin in µBR, demonstrated in Fig. 6F,G.

RA can penetrate the stratum corneum layer and, to a small extent, into the dermis because they are fat-soluble^48^. To evaluate the transport of RA inside the skin in the chip, diffusion was measured. As shown in Fig. 6G, the accumulation of RA in micro molar scale is detectable and through integration of the mass, the time period the total mass transfer per certain area of the human skin before delivery to vasculature is measurable.

Our findings not only supported the integrity and coherence of the cultured biopsy skin structure in the µBR (ex-vivo situation), but also the deterioration results in TCPs samples suggested that the traditional skin model studies should be replaced by on-a-chip tests due to the lack of accuracy in traditional TCPs cultures.

## Conclusion

A µBR as a skin model was designed and fabricated. Normal full-thickness human skin biopsy was implanted in parallel with the tissue culture in TCPs. The two groups were compared and evaluated through several assays, live/dead, MTT, SEM, IHC, RT-PCR, tensile strength, and swelling ratio. The experiments revealed that keeping the biopsies in the µBR can retain different properties of the skin more like the native skin in comparison with TCPs. On the flip side, traditional TCPs culture caused dramatic changes in the skin in seven days leading to unrealistic results in studies. Finally, a sample of transdermal drug diffusion measurement was performed to illustrate a real application of the fabricated microdevice.

## Materials and methods

### Design and fabrication of microbioreactor

The pumpless µBR for the skin-on-a-chip model was designed in Corel (version. 21) and prepared with polydimethylsiloxane (PDMS) via the soft-lithography method. The master mold was prepared with SU-8 photoresist.

The structure of the skin chip consists of two layers. The bottom layer, which is named the skin chamber containing microchannels and the top layer which is built with two 10 mm diameter cylinders as inlet and outlet and a square shape as a location for implanting and cultivating a living biopsy skin. The diameter of the culture chamber was 8 × 8 mm and the width and height of the microchannel were 50 µm. The dimensions of the two PDMS layers were 25 × 75 mm. A 10 × 10 mm of human skin biopsy is mounted on bottom layer of PDMS then the top layer of PDMS sits on the skin so that the PDMS layers sandwich the skin. The two-layer PDMS was sealed with a two-layer polystyrene sheet on top and bottom tightened with screws. Sterilization of the chip was done using ethanol wash and rinsed with PBS.

The bag hangs from the tube and the tube is connected to the bottom of the bag. Its contents "drop by drop" enter the tube at a specific time interval. The tube leads to a hollow needle attached to the inlet. The perfusion rate can be controlled by a roller clamp. The chip system works because gravity moves the medium through the tubes to the skin chamber and the gravitational pressure on the medium helps it go down the tube. Every 8–12 h based on the pH changes in the µBR medium, the medium (High glucose DMEM, enriched with 10% FBS & 10% Goat serum) is changed through withdrawal of 500 µL of the medium from outlet and adding the same amount of fresh medium dropwise into the inlet.

The pH-change is defined through the medium color change from bright pink to yellow, based on pH color indicator in the medium.

The medium was added to the top chamber and passed through the channels to the outlet using capillary forces and the height pressure initiated from the medium height gradient between the inlet and outlet. The culture medium diffused through simulated vascular microchannels.

### Tensile testing

The tensile resistance assessment, known as the stress test, was performed to resemble the degree of deterioration of the skin over time. Tensile strength (UTS) and elongation (EL) can be achieved specifically when the material is stretched. To evaluate the tissue structure, the skin was cut at 0.4 mm thickness. After placing the skin sample in the machine (Santam, Iran), a tensile force (10 kg) was applied to the sample until failure occurred. The force required to create the elongation was reported and the force–elongation curve was plotted.

### Swelling ratio

The skin can absorb and secrete substances based on the body's needs and it is selectively permeable to specific chemicals. The amount of water absorbed by the skin was assessed for 32 h.

First, the initial weight of the skin samples was measured, then samples were placed in a culture medium and weighed after 15 and 30 min, 1, 2, 4, 8, 16, and 32 h.

### Skin-on-a-chip culture

Skin tissue was obtained from patients undergoing graft surgery with an age range of between 20 and 40 years in Motahari hospital. All methods in this study were approved by the Research Ethics Committee of the Science and Research Branch, Islamic Azad University of Medical Sciences (IRI.IAU.SRB.REC.1400.172). Epidermal and dermal layers were dissected from subcutaneous as full-thickness skin grafts using a dermatome (Fig. 1A). The samples were maintained in PBS at 4 °C until culturing in the laboratory. Fresh skin was cultured on the same day of isolation. Skin samples were spread out on a petri dish and cut in 1 × 1 cm^2^ with 0.4 mm thickness. Afterward, the samples were washed with PBS containing 0.02% gentamicin antibiotic. Skin sections were implanted in a microbioreactor (µBR) with the outer skin surface facing upwards (Fig. 1B), cultured in a tissue culture plate (TCP) in two groups, and incubated at 37 °C, 5% CO_2_, and 95% humidity.

Tissue samples were cultured in DMEM medium, enriched with 10% FBS and 10% Goat serum containing 1% penicillin/streptomycin. The culture medium in the µBR was changed twice a day through withdrawal from the outlet and adding fresh medium to the inlet. The medium was changed every day in TCPs group with the same medium composition used in the other group.

### Full thickness skin viability assay

The viability of the tissue sample was evaluated through Acridine orange (AO) staining (Merck) and MTT (Thiazolyl Blue Tetrazolium Bromide) assay (Sigma Aldrich, UK) in both groups (after inserting the skin samples in the µBR and TCP). The culture medium was changed twice a day.

For AO staining and after fixation by 4% paraformaldehyde, the samples were embedded in paraffin and sectioning were performed in two groups. After deparaffinization, samples were immersed in the dye for 5 min. Then the samples were washed with PBS and images were captured by fluorescent microscope (BioTek, USA).

To perform MTT assay, after culturing of the skin samples in the TCP and µBR for 0, 1, 3, and 7 days, MTT solution was added and incubated for 3 h. The sediments dissolved in dimethyl sulfoxide. Samples were shaken for 15 min in the dark condition. After discarding the samples, the absorbance of the extracted solution was measured at 570 nm using an ELISA reader instrument (Epoch, BioTek, USA). Finally, µBR and TCP data were compared (n = 3).

### Histological staining

The functions of skin tissue are tightly related to its histological structure. To analyze the morphology of the skin structure, the samples were subjected to hematoxylin and eosin staining. First, the skin samples were cultured in the µBR, and TCPs were fixed in 4% formalin. Then the samples were embedded in paraffin. After sectioning on glass slides with a thickness of 5 µm in two groups (µBR and TCP), for H&E staining, the samples were deparaffinized in xylene and then rehydrated in decreasing ethanol concentrations. Finally, the samples were stained with H&E and Masson's-trichrome staining following the manufacturer's protocol.

### Scanning electron microscopy (SEM)

Skin samples in both groups (µBR and TCPs) were fixed in 4% glutaraldehyde for 3 h. For dehydration, a gradual descent series of ethanol in distilled water was used for 15 min on each sample (60, 70, 80, 90, 96, 100%). The samples were coated with a gold layer using a gold sputter and images were captured by SEM (FEI, USA).

### RT-qPCR

To evaluate skin graft gene expression, total RNA was extracted by RNA Extraction kit (RiboEx, GeneAll Biotech, Korea) in the µBR and TCP samples on days 0 and 7. Total RNA concentrations were determined using a NanoDrop and the absorbance ratio was measured at 260 nm (Thermo Scientific, USA). Complementary DNA (cDNA) was synthesized according to the manufacturer’s instructions (cDNA synthesis, GeneAll, Korea). Then RT-qPCR (RT-qPCR kit, Takara) was performed to analyze the expression of *αSMA* (as a Fibrotic gene), *OCT-4* (as a Proliferation gene)*, BAX* (as an Apoptotic gene), *CK18* (as a Dermal specific gene) and –ACTIN as a housekeeping gene. Primers for RT-qPCR are designed for specific targets. PCR primers were designed, and specificity was confirmed with BLAST. The sequences of the primers are mentioned in Table 1. All experiments were performed in duplicate, and the results of qRT-PCR were analyzed by REST software. The amount of gene expression was calculated by the fold change formula.

**Table 1:** List of primers.

No	Gene name	Sequence (5′–3′)
1	*CK 18*	Forward: TGCTGCTGATGACTTTAGA
		Reverse: TTACTTCCTCTTCGTGGTT
2	*αSMA*	Forward: GACGCACAACTGGCA
		Reverse: GCAGTAGTAACGAAGGAATA
3	*OCT4*	Forward: CGCCGTATGAGTTCTGT
		Reverse: GGTGATCCTCTTCTGCTT
4	*BAX*	Forward: CAAACTGGTGCTCAAG
		Reverse: CACAAAGATGGTCACGG
5	*β-ACTIN*	Forward: CTTCCTTCCTGGGCATG
		Reverse: GTCTTTGCGGATGTCCAC

### Immunohistochemistry analysis

First, the skin tissue samples were fixed in 4% paraformaldehyde (Merck, Germany) for 3 h, at room temperature. Next, the samples were washed with PBS and processed for paraffin embedment. After sectioning on four separate glass slides and deparaffinized, Rehydrated samples were blocked with 3% BSA for 1 h and then primary antibodies [1:100 diluted], VIMENTIN (Abcam), E-CADHERIN (Abcam), PANCYTOKERATIN (zymed) and CYTOKERATIN 18 (Abcam) were used in a humidified chamber at 4 °C overnight. The samples were washed with PBS containing 0.025% tween 20 (PBST) and they were incubated with secondary antibody Alexa Fluor 555 (goat anti-mouse, Invitrogen) for 1 h. The samples were washed with PBST and stained with DAPI for 5 min. Finally, immunofluorescence images were captured using fluorescent microscope (BioTek, USA) on days 0, 1, and 7.

### Transdermal drug delivery study

To evaluate mass transfer from the skin, the diffusion rate of the drug was measured through the skin layers. The retinoic acid (RA) calibration diagram was drawn in 1 mM, 500 µM, 250 µM, 125 µM, 65 µM, 35 µM, 15 µM, and 10 µM concentrations diluted in a cell culture medium. First full thickness skin graft was placed in the skin chamber and infused medium from inlet. Then 10 µl of the 1 mM concentration of RA (Sigma Aldrich) was applied on the top surface of the skin implanted in the µBR cultivation chamber. Sampling was performed from the outlet part from zero to 8 h at 30 min intervals. 20 µl of medium was collected from outlet and added the same amount of new medium in inlet afterward. In addition, the permeability was analyzed by a spectrophotometer (Shimadzu, Japan) at 340 nm.

### Statistical analysis

All statistical analyses were performed in SPSS (Version 23). After the normality test, data were analyzed with parametric tests (one-way ANOVA) and to compare Macro and microsystems, paired student t-test was applied, and the results were reported as mean ± standard deviation. The experiments were performed in triplicate (*p* ≤ 0.05).

### Ethical approval and consent to publish

Skin tissue biopsies were obtained from patients undergoing graft surgery in Motahari hospital. Informed consent was obtained from all patients for biopsies. This study was conducted in accordance with the principles of the Islamic Azad University of Medical Sciences, and it has received approval from the Ethics Committee of the Science and Research Branch, Islamic Azad University of Medical Sciences under IRI.IAU.SRB.REC.1400.172 permission.

## Supplementary Information

Supplementary Information.

## Data Availability

All data generated or analyzed during the current study are included in the manuscript. The data of this study is available on reasonable request from the corresponding author.
