# Supplementary material for: Design and evaluation of a skin-on-a-chip pumpless microfluidic device
Source: Sci Rep. 2023 May 31;13:8861. doi: 10.1038/s41598-023-34796-3 (PMC10232512; doi:10.1038/s41598-023-34796-3)
Supplement: Supplementary file 1 — Supplementary Information. [file 41598_2023_34796_MOESM1_ESM.pdf]

Design and evaluation of a skin-on-a-chip pumpless microfluidic device

Marjan Mohamadali[1], Ali Ghiaseddin [2,3,4*], Shiva Irani[1], Mohammad Amir Amirkhani[5], Mostafa Dahmardehei[6]

[1]Department of Biology, Science and Research Branch, Islamic Azad University, Tehran, Iran,

[2] Department of Chemistry, Michigan State University, East Lansing, MI, USA,

[3] Institute for Stem Cell Research and Regenerative Medicine, Tehran University of Medical Sciences, Tehran, Iran,

[4] Department of Anatomical Sciences, Faculty of Medical Sciences, Tarbiat Modares University, Tehran, Iran,

[5] Skin and Stem Cell Research Center, Tehran University of Medical Sciences, Tehran, Iran,

[6] Department of Plastic and Reconstructive Surgery, Burn Research Center, Iran University of Medical Sciences, Tehran, Iran.

Microbioreactor Images

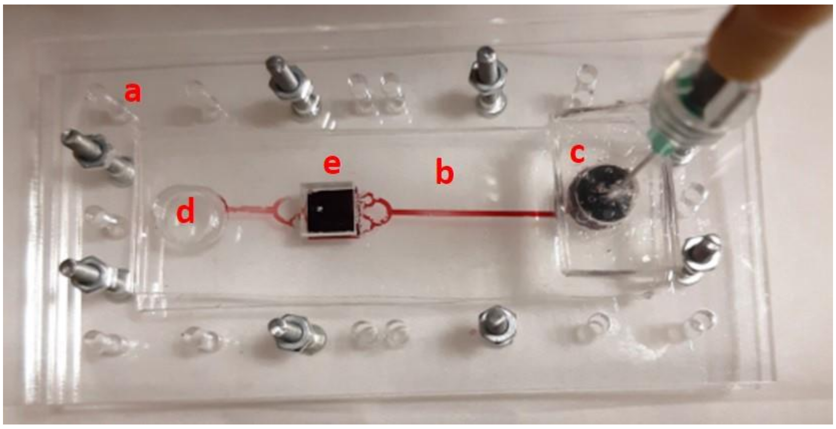

Fig 1S. A) The image shows an assembled device running with red dye, a: Plexiglas housing, b: PDMS top layer, c: Inlet, d: outlet, e: µBR culture chamber.

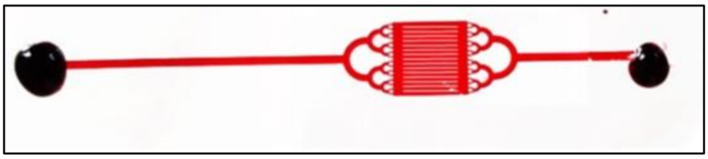

Fig 1S. B) The image shows the bottom layer PDMS of the device run with dye. The location of the inlet, outlet and µBR chamber with feed distributors and the capillaries can be find.

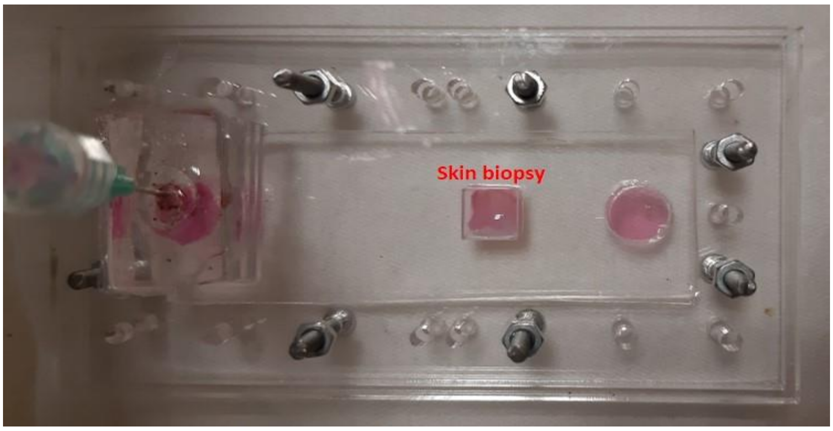

Fig 1S. C) The image shows the μBR with skin biopsy in place and running with perfusion.

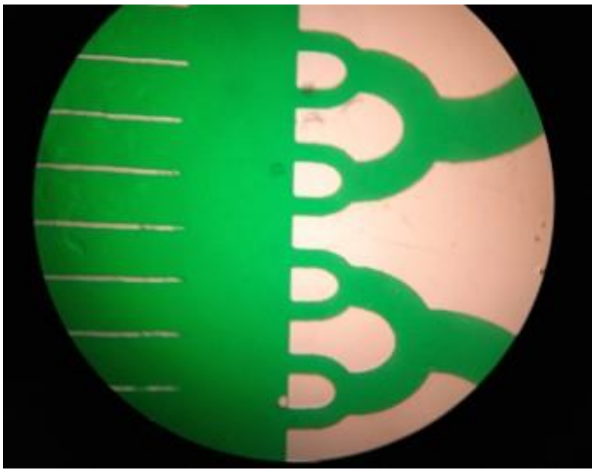

Fig 1S. D) The image shows a microscopic image of the entrance of the μBR chamber, Feed distributers and the capillaries are visible with green dye.
